# Cumulative Environmental Vulnerability and Environmental Justice in California’s San Joaquin Valley

**DOI:** 10.3390/ijerph9051593

**Published:** 2012-05-03

**Authors:** Ganlin Huang, Jonathan K. London

**Affiliations:** 1 Center for Regional Change, University of California at Davis, 152 Hunt Hall, One Shields Avenue, Davis, CA 95616, USA; 2 Department of Human and Community Development, University of California at Davis, 2335 Hart Hall One Shields Avenue, Davis, CA 95616, USA; Email: jklondon@ucdavis.edu

**Keywords:** cumulative environmental hazards, social vulnerability, environmental justice

## Abstract

The identification of “environmental justice (EJ) communities” is an increasingly common element in environmental planning, policy, and regulation. As a result, the choice of methods to define and identify these communities is a critical and often contentious process. This contentiousness is, in turn, a factor of the lack of a commonly accepted method, the concern among many EJ advocates and some regulators that existing frameworks are inadequate, and ultimately, the significant consequences of such designations for both public policy and community residents. With the aim of assisting regulators and advocates to more strategically focus their efforts, the authors developed a Cumulative Environmental Vulnerability Assessment (CEVA). This CEVA is composed of a Cumulative Environmental Hazard Index and a Social Vulnerability Index, with a Health Index as a reference. Applying CEVA produces spatial analysis that identifies the places that are subject to both the highest concentrations of cumulative environmental hazards and the fewest social, economic and political resources to prevent, mitigate, or adapt to these conditions. We recommended that these areas receive special consideration in permitting, monitoring, and enforcement actions, as well as investments in public participation, capacity building, and community economic development.

## 1. Introduction

The concept of cumulative impacts is increasingly in evidence in public policies, regulation, and advocacy in the domains of public health and environmental quality [1,2,3]. In contrast with environmental and health science and policy that assess risks from exposures to individual contaminants, often through one media and at one point in time, a cumulative impacts approach considers the experience of encounters with multiple environmental hazards through multiple media and over time [1,2,3]. The cumulative impacts approach also considers the social, economic and political context of these exposures, and ways in which these factors may exacerbate the vulnerability experienced certain places and populations to environmental hazards [4,5,6,7,8]. Recent pioneering work offers a sophisticated set of environmental justice indices and screening methods to map and prioritize the most vulnerable communities for interventions to improve current conditions and prevent future harm. Some of these innovations have been adopted by public agencies at the state and federal levels [9,10,11,12,13]. This social-environmental analysis is illustrated in a recent policy guidance document from the California EPA, in which cumulative impacts are defined as, “The exposures, public health or environmental effects from the combined emissions and discharges, in a geographic area, including environmental pollution from all sources, whether single or multi-media, routinely, accidentally, or otherwise released. Impacts will take into account sensitive populations and socio-economic factors, where applicable and to the extent data are available.” [14].

Advocates for environmental justice have turned to cumulative impacts as a powerful way to document their experiences of confronting environmental hazards in the places in which they “live, work, learn, and pray” [15] and that often affect low-income populations and communities of color in disproportionate patterns [16,17]. They point to a “double jeopardy” of environmental injustice in which the people with the fewest social, economic and political resources experience the greatest concentrations of environmental threats to their health and well-being [18] In particular, factors such as historical patterns of housing segregation, limited political and social capital, and policy processes that are inaccessible by grassroots constituencies have been shown to combine to create a “risks-cape” that systematically places communities with lower incomes and greater concentrations of people of color in harm’s way from a wide range of environmental hazards [18,19,20,21]. In the face of these conditions, activists call for three dimensions of environmental justice: distributional justice for addressing disproportionate burdens of environmental hazards and the absence of environmental goods, procedural justice for meaningful access to decision-making, and cognitive justice to consider local knowledge legitimate in the assessment and mitigation of environmental hazards [22,23].

This case study of California’s San Joaquin Valley (SJV) seeks to contribute to the science and policy of cumulative impacts and environmental justice by adapting leading existing analytical methods to fit the geographic profile of this diverse region. The purpose of this study was to assist regulators and advocates to more strategically focus their efforts on the places confronting the highest levels of environmental hazards and the highest levels of social vulnerability. We developed a Cumulative Environmental Vulnerability Assessment (CEVA) including a Cumulative Environmental Hazard Index (CEHI), a Social Vulnerability Index (SVI) and a Health Index (HI). 

## 2. Study Site

**Figure 1:**
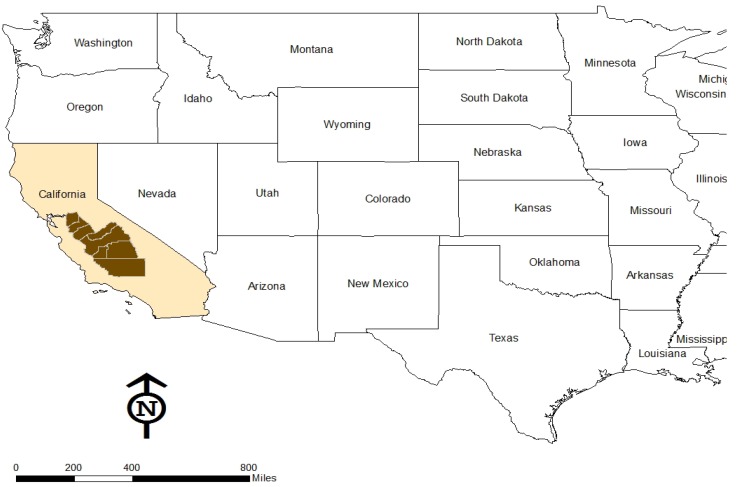
Study area: San Joaquin Valley Region.

The SJV is the southern half of California’s 450-mile-long Central Valley, an elongated “bowl” bounded on three sides with mountains and extending through the heart of the state (Figure 1). It has eight counties: San Joaquin, Stanislaus, Merced, Madera, Fresno, Tulare, Kings, and Kern. The SJV has a population of 4.2 million, of which 46% are Latino/Hispanic residents, many of whom are first generation immigrants from Mexico and elsewhere in Latin America. The ethic mosaic of the region is also made up of immigrants from South East Asia, South Asia, Europe, as well as historical settlements of Native Americans, African Americans, and Caucasians (many of the latter from the Dust Bowl migration). The region is considered politically conservative, with strong property rights, pro-business and limited government values, although a new generation of progressive political leaders, is beginning to change this profile [24].

The SJV is world-renowned for its rich agricultural bounty, with three of its counties (Fresno, Tulare, Kern) occupying the top ranks of agricultural production in the country. Large-scale irrigation works, intensive chemical inputs (pesticides, fertilizers), and a labor market dependent on a low-wage work force have driven the richness of the agricultural sector but also the poverty of many who work the land, and the contamination of its air and water [25]. Sometimes called the “other California” and compared to Appalachia, the San Joaquin Valley is a land of “poverty amidst prosperity” with concentrated poverty and associated social ills despite the wealth of its agricultural industry [25,26,27,28].

Coupled with significant goods movement infrastructure such as highways and freight lines, industrial developments (refineries, manufacturing, power plants, and waste disposal sites) as well as the bowl-like geo-morphology, the SJV is considered to have among the worst air quality in the nation [29,30]. While important progress has been made by environmental regulators at the regional (San Joaquin Valley Air Pollution Control District), state (California EPA/Air Resources Board), and federal levels (US EPA), the SJV is in non-attainment for criteria air pollutants (*i.e*., ozone and particulate matter). Within this regional non-attainment area, some communities, particularly in the southern end of the bowl have extremely high air quality impacts [29]. Residents of the San Joaquin Valley suffer from high rates of asthma and other respiratory ailments [29,30,31]. Madera, Fresno, and Kings Counties for example, have rates approximately twice that of the state as a whole for asthma-related emergency room visits by young children (ages 0–4) [32]. According to one recent study, the economic benefits of the region meeting air quality standards for ozone and particulate matter would top $6 billion per year in reduced health, missed work and school, and premature death; this is equivalent to a payment of $1,600 per person per year [33]. In addition, residents in many of the rural unincorporated communities often suffer significant impairment of their drinking water supplies [34,35]. In sum, living near freeways and rail lines, working in outdoor occupations with inadequate safety precautions, drinking polluted water, and lacking access to affordable and healthy food, health insurance, and quality medical care together create a spatially and racially patchy “riskscape” [18].

Mobilized by these multiple environmental and social threats, a vibrant social and environmental justice movement has organized in the SJV, with roots back to the International Workers of the World in the 1920s and 1930s, to the United Farm Workers in the 1960s and 1970s, to the contemporary environmental justice movement beginning in the 1980s and continuing to today [36,37,38,39,40]. Many of these movements have organized on a regional scale in attempts to address the “spatial mismatch” between the local sites of impact from environmental hazards and the wider-scale drivers of these phenomena [41,42]. One prominent example of this multi-scalar organizing is campaigns that focus pressure on the regional San Joaquin Valley Air Pollution Control District and its nested organization with state and federal regulatory agencies. 

We therefore selected the eight-county SJV as a strategic case study of the complex relationship among cumulative environmental risks, social vulnerability and health, and to offer this methodology to public agencies and advocates working to improve health conditions in the region. 

## 3. Methods

We developed CEVA for the eight-county SJV region. This process involved a collaborative research model where a university-based team drew on the leading methods from the academic and professional literature and partnered with a coalition of environmental justice and health advocates who contributed their sophisticated knowledge of local and regional environmental, social, economic, and political contexts. Through a three-year iterative process, the combined team developed the CEVA framework [43,44].

The CEVA is composed of a Cumulative Environmental Hazard Index and a Social Vulnerability Index, with an additional Health Index as a reference layer. Applying this CEVA produces spatial analysis that identifies the places that are subject to both the highest concentrations of cumulative environmental hazards and the fewest social, economic and political resources to prevent, mitigate or adapt to these conditions. Table 1 presents the indicators for all three of these indices. 

**Table 1:** Cumulative environmental hazards index, social vulnerability index and health index.

Index	Dataset	Source	Time
Cumulative Environmental Hazards Index	Toxic release inventory sites	U.S. EPA	2006
	Refineries	U.S. EPA	2006
	Hazardous waste treatment, storage and disposal facilities	U.S. EPA	2006
	Chrome platters	U.S. EPA	2006
	Total amount agri. pesticide application per 1 mile^2^	CA Dept. of Pesticide Regulation	2007
	National-scale air toxic assessment	U.S. EPA	2005
Social Vulnerability Index	Percent of people younger than 5 or older than 60	American Community Survey (ACS)	2006–2009
	Locations of health care facilities	Cal-Atlas	2010
	Percent of linguistically isolated households	ACS	2006–2009
	Percent of population in poverty	ACS	2006–2009
	Percent of people of color	ACS	2006–2009
	Percent of people older than 25 without a high school diploma	ACS	2006–2009
Health Index	Low birth weight rate	CA Dept. of Public Health	1999–2007
	Years of potential life lost before age 65	CA Dept. of Public Health	1999–2007
	Asthma hospitalization rate ages 0–19	CA Office of statewide health planning and development	1999–2007

### 3.1. Cumulative Environmental Hazard Index

The CEHI is a relative measure of environmental hazards in and around each block group with possible scores between 0 and 1. The higher the value, the greater the concentration of environmental hazards within or around the block group. The CEHI was calculated at the census block group level from the following six datasets: toxic release inventory sites, refineries, hazardous treatment, storage and disposal facilities (TSDs), chrome platters, pesticide application, and the national-scale air toxic assessment (NATA). These indicators were selected based on both leading cumulative impact methods [1,2,3,4,5,6,7,8,9,10,11,12,13] and input from the project’s environmental justice partners as their most pressing concerns. The first four datasets, toxic release inventory sites, refineries, hazardous TSDs and chrome platters, are all point source data, indicating the specific location of the pollution sites. These four datasets were merged into one file and a 1-mile buffer zone was drawn around the points in ArcGIS 9.3^TM^. The percent area of each block group that falls within the 1-mile buffer was calculated, to be incorporated into the CEHI. Here we focused on the percent area of each block group falls within the 1-mile buffer instead of the numbers of the pollution sites to avoid double-counting. Many pollution sites were listed in multiple inventories. Giving weight on the number of pollution sites within each block group may count the same pollution site multiple times. The percent area falls within the buffer zone presented the magnitude impacted by the point source pollution sites. We realized considering the percent area falls within the buffer zone underestimated the impact by the point source pollution sites, which will be discussed as one of the limitations of CEVA. 

Pesticides application density, of active ingredients, was based on the public land survey system, which divided land into sections with an approximate 1-square-mile area. To break down these large-spatial units to a scale in line with potential individual exposures, we divided each section in ArcGIS 9.3^TM^ into 16 units with an approximate size of 100 m × 100 m and assigned each unit the pesticide density from the section where the unit was located. Then we calculated pesticide density for each block group as the mean value of that from all the units within or including at least 50% coverage of the block group. 

National air toxic assessment (NATA) estimates the risk of different kinds of cancer and other serious non-cancer health effects from inhaling air toxics. We used the latest available data from the 2005 NATA in this study. NATA uses census tracts, which is one level higher than block group as the unit of analysis. In the San Joaquin Valley, one census tract contains an average of 5.5 block groups. We assigned the total risk of cancer of a tract to all the block groups that were contained within it. Although assigning the same score of a tract to all the block groups within it did not consider the air toxics variation among the block groups, it preserved the precisions of the source data sets (*i.e*., tract for NATA and block groups for the rest data sets) the best while incorporated them into CEHI.

Lastly, we normalized the percent area of each block group within 1-mile buffer of point source pollution, pesticides density and total risk of cancer by dividing each value by the maximum value of the dataset, and calculated the mean value of the three normalized datasets. In order to have the scores spread out widely and range between 0 and 10, we normalized the mean values by dividing its maximum value and multiplying the entire data set by 10 to generate the CEHI value for each block group. The formula calculating CEHI is shown below in two steps:


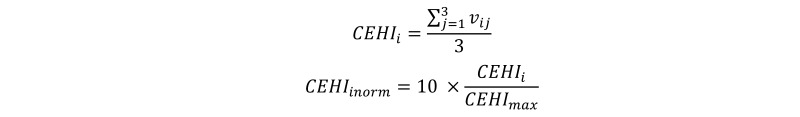


where *CEHI_i_* is the cumulative environmental hazard index score for block group *i* before normalized;

*v_i1_* is the percent area of block group *i* that falls in the 1-mile buffer of point source pollution sites;

*v_i2_* is the normalized total risk of cancer for block group *i* from NATA;

*v_i3_* is the normalized pesticide application for block group* i*;

CEHI_max_ is the maximum value of *CEHI_i_* of all the block groups in SJV;

*CEHI_inorm _*is the normalized cumulative environmental hazard index score for block group *i*.

### 3.2. Social Vulnerability Index

The SVI was developed to describe the sensitivity of the community to health challenges and resources to mitigate negative health impacts from environmental hazards. The SVI is a relative measure with values between 0 and 1: the higher the value, the more vulnerable the residents of a block group are to the effects to environmental and other hazards. The SVI was calculated at the census block group level from the following six datasets: locations of health care facilities, poverty rate, education, linguistic isolation, race/ethnicity, and age. These indicators were selected based on a combination of leading CI studies and the input of community partners, as the factors their increase sensitivity to hazards (age, residence in in-patient care facilities) or decrease the social, economic and political capital needed to effectively prevent or mitigate hazards (poverty, education, racial/ethnic concentration, language ability.)

In this study we consider the presence of in-patient health care facilities within given block groups as an indicator of the presence of sensitive receptors to environmental hazards. While health care facilities can also be an asset to mitigate negative environmental impacts, this positive function was not considered here because the presence of a health care facility in or around a community may not indicate that it is accessible or utilized by local residents [9]. We retrieved the dataset of locations of health care facilities from the Cal-Atlas website. In ArcGIS 9.3^TM^, we drew a 1-mile buffer zone around each health care facility and then calculated the percent area of each block group within the buffer zone. We calculated the mean value of the percent area of each block group within the buffer zone, percent population in poverty, percent people older than 25 without a high school diploma, percent household that are considered linguistically isolated (defined as without a member older than 14 speaks English fluently), percent people of color (other than non-Hispanic White) and percent population older than 60 and younger than 5. Finally, we normalized the datasets by dividing its maximum value and multiplying by 10 to generate the SVI value for each block group. The formula of calculating SVI is shown below:


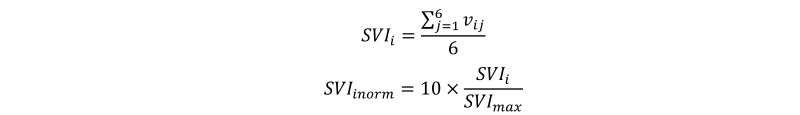


where *SVI_i_* is the social vulnerability index score for block group *i* before normalized;

*v_i1_* is the percent area of block group *i* that falls in the 1-mile buffer of health facilities;

*v_i2_* is percent population in poverty for block group* i*;

*v_i3_* is percent people older than 25 years old without a high school diploma for block group* i*;

*v_i4_* is percent linguistically isolated households for block group* i*;

*v_i5_* is percent people of color for block group* i*;

*v_i6_* is percent people older than 60 or younger than 5 for block group* i*;

*SVI_max_* is the maximum value of *SVI_i_* of all the block groups in SJV;

*SVI_inorm_* is the normalized social vulnerability index score for block group* i*.

### 3.3. Health Index

We constructed HI from data in low birth weight rate, years of potential life lost before age 65 (YPLL65) and asthma hospitalization rate ages 0–19. These indicators were selected to represent conditions across the life span, and as those that can serve as proxies for overall health status. Due to data availability, health data is based on zip code. We first normalized low birth weight rate, YPLL65 and asthma hospitalization rate ages 0–19 by dividing the data sets by its maximum value. For each zip code, the maximum value of the three health indicators was assigned as the value of health index. In this way, the health index was designed to reflect high value (*i.e*., health problems) from any indicator. Then we converted the unit of analysis from zip code to block group. We generated a raster file in ArcGIS 9.3^TM^ with a cell size of 100 m × 100 m. Each cell was assigned the value of HI from the zip code where the cell was located within. Second, we calculated the mean value of the HI from the raster file for each block group. Similarly to the way we converted tract-level NATA data to block group level, such conversion preserved the original precision of the source datasets. However, it is worth noting that such “block group level” NATA and health data are generated as a middle step and to be incorporated into CEHI and HI along with other datasets at block group level. The precisions of NATA and health are still tract and zip code respectively. The raster technique did not improve the precision of the original datasets. The formula of calculating HI is shown below:


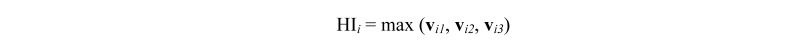


where HI*_i_* is the Health Index score for block group i;

**v***_i1_* is normalized incidence of low birth weight for block group i;

**v***_i2_* is the normalized YPLL65 block group i;

**v***_i3_* is the normalized hospitalization rate for asthma (ages 0–19) for block group i.

## 4. Findings

**Figure 2:**
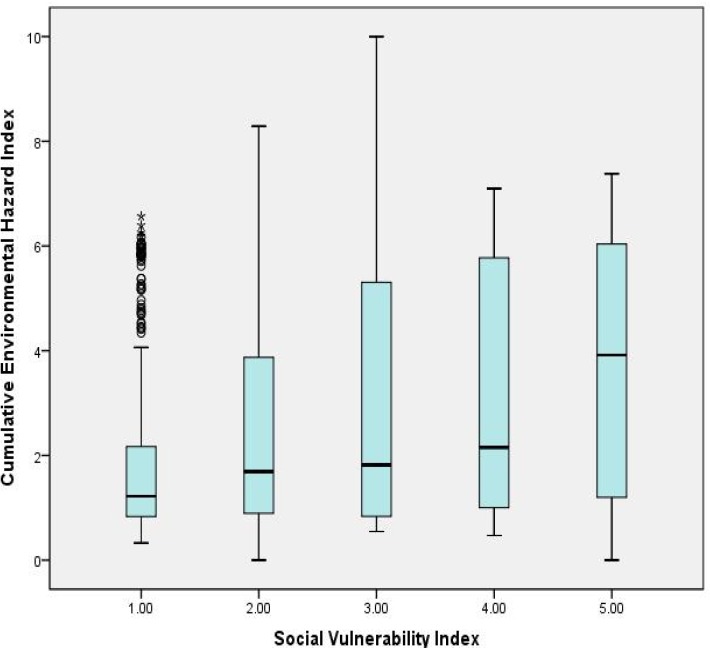
Box plot of CEHI and SVI.

We generated a boxplot to present the distributions of CEHI within the five quintile groups of SVI (Figure 2). The boxplot presents the five statistics (minimum, first quartile, median, third quartile and maximum) within each category. Each category contains about 450 cases. The results show an increase of the median CEHI when the SVI increases across the five social vulnerability index categories.

Using the Pearson’s statistic, we determined that the CEHI is significantly correlated with the SVI (0.296) and, to a lesser but still significant degree with the HI (0.092). This statistic also shows that the SVI is significantly correlated with the HI (0.231). There was not a statistically significant correlation between the CEHI and the HI, for reasons considered below. The Pearson’s correlation matrix is presented in Table 2.

**Table 2:** Pearson’s Correlation Matrix of CEHI, SVI and HI.

		CEHI	SVI	HI
CEHI	Pearson Correlation	1	0.296 **	0.092 **
	Sig. (2-tailed)	-	0.000	0.000
	Block Groups (n)	2,237	2,237	2,237
SVI	Pearson Correlation	0.296 **	1	0.231 **
	Sig. (2-tailed)	0.000	-	0.000
	Block Groups (n)	2,237	2,241	2,241
HI	Pearson Correlation	0.092 **	0.231 **	1
	Sig. (2-tailed)	0.000	0.000	-
	Block Groups (n)	2,237	2,241	2,241

** Correlation is significant at the 0.01 level (2-tailed).

### 4.1. Spatial Relationships between CEHI, SVI, and HI

**Figure 3:**
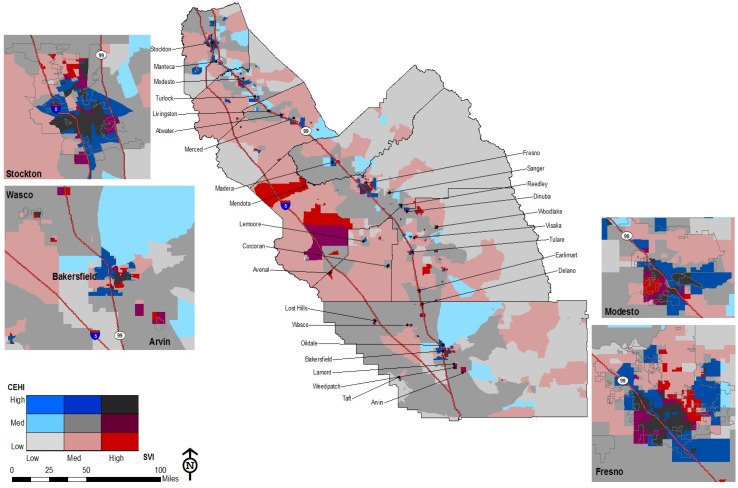
CEVA for the San Joaquin Valley.

Arraying the CEHI and the SVI in one map produces a patchwork spatial pattern for the region in which where one lives has a profound effect on the environmental hazards in the proximate risk-scape (Figure 3). 

**Figure 4:**
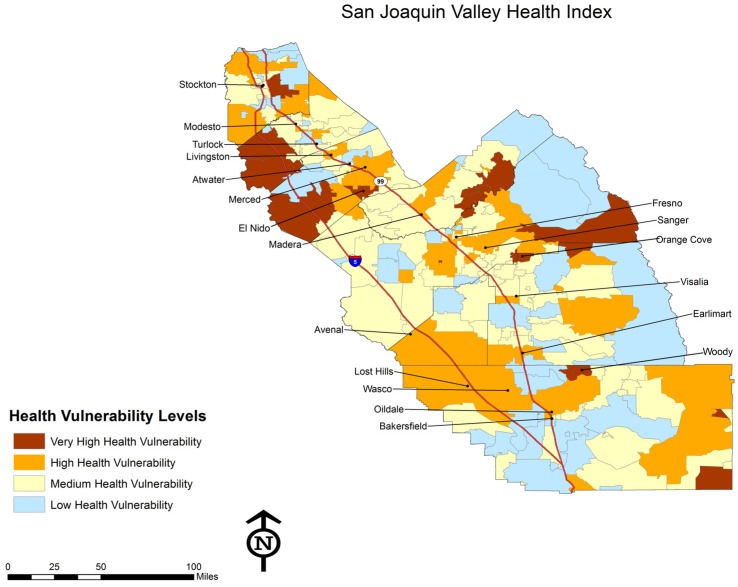
Health Index.

The map of CEVA reveals two important patterns. First, in urban areas with the highest levels of environmental hazards and social vulnerability, there is a patch work pattern of separate and unequal places. Second, significant overlap between environmental hazards and social vulnerability also occurs in many small, rural towns throughout the region where low-income communities and communities of color live amidst agricultural fields with intensive pesticide applications and non-agricultural industries such as power plants and waste disposal facilities. Our analysis also examined the spatial distribution of health conditions with potential environmental influences. This is represented in Figure 4 below.

Health conditions are caused by a wide range of factors, including genetics, individual behaviors, health care, and the social and physical environment. This study addresses only the issue of social and physical environment, and therefore does not provide a comprehensive analysis of the breadth of causes of health problems in the region. However, using the Health Index, this study demonstrates that there are many thousands of people living with severe environmental hazards and high social vulnerability who are contending with a range of health problems that other research has shown to be correlated with environmental and social stressors. This is seen especially in rural areas in the northwest section of the region, with some pockets along the foothills on the eastern side of the valley. 

## 5. Limitations

There are some limitations of our model for the three indices–CEHI, SVI, and HI. To begin, our model is only as accurate as the source data sets. All the data sets we used are generated either on national or state scale and publicly available, which allows our model to be replicated. These data sets are the most reliable ones in their field. However, restricted by time and other resources, these data sets all have their own limitations, which are published along with the data sets. For example, NATA does not consider diesel when assessing cancer risk. The health impact from diesel may be addressed with transportation volume data, which is our next step for this study.

Second, there are certain issues—including those correlated with severe health conditions—that lack data sets that are reliable and comprehensive in geographic scale. Water quality, for example, has long been an issue in the San Joaquin Valley region and could potentially have very important health impacts on residents. While there is some water quality data available, it is not available at the census block group for the region as a whole, and therefore is not possible to incorporate this data into the CEHI. 

Third, the indices use data sources that cover a range of stages in the emissions, potential exposures, toxicity, and health risk process. For example, the point source data (e.g., TRI, TSDs) indicate only the presence of an emitting facility, but not the amounts, the fate or the toxicity of the pollutants. The emissions data (e.g., pesticide application) indicates the amounts, but not the fate, exposures or toxicity of the substances. Our CEHI also provides a binary (presence/absence) for the location of TRI and other point sources, as opposed to a score that incorporates the density of such sources in a given area. This is one of the factors that suggest that the CEHI is an under-estimate of the concentrations of environmental hazards in any given block group.

Fourth, our model is limited by its geographic unit of analysis: the census block group, which is the smallest geographic unit that the U.S. Census Bureau aggregates certain demographic data. Therefore, CEHI and SVI calculated from our model are mostly likely to be accurate when we look at areas that are larger than a block group. Our model cannot provide much reference to areas smaller than a block group.

The fifth limitation is related to using block group as the unit of analysis. By doing this, we assumed that people spend most of their time in or nearby their home and spread out evenly in a block group. In fact, people constantly move and population densities often vary greatly within each block group. Using residential areas as the unit of analysis can address the variation of population densities. However, the prevalence of unauthorized settlements in rural areas makes it likely that there are people living in areas not included in “residential” land use zones. A final caution on the use of place-based analysis such as the CEHI is that it does have a limited ability to account for hazards that originate outside the block group (e.g., regional flows of air pollution). The inclusion of the NATA data does partially address this. 

Finally, the indices are relative to the region and not absolute measures. Relative indices present the regional patterns well but preclude being able to track progress over time, as conditions in individual places or the entire region may have declined. Because the SJV faces high incidence of environmental and social problems relative to the state and nation as a whole, the relative low or medium score of some areas (compared with SJV) can mask vulnerabilities. 

More broadly it is important to state that the CEHI is not a formal risk assessment that quantifies the specific pollution exposures. We used metrics that measure emissions, potential exposures, as well as health risks. It should also be noted that there are a range of other data sets that were not included in this index because of challenges of data availability at the appropriate spatial scale, with a region-wide scope, or with reliable sources. Therefore, the CEHI should be understood as a screening method, helping to identify places with higher relative degrees of environmental hazards compared to the region as a whole. 

Future improvements to the CEVA ought to include strategies to address the above limitations, with a priority on including air quality (PM, Ozone), drinking water quality (particularly nitrate and arsenic) and transportation volume; integrating measures of pesticide and point source site emissions fate and toxicity; incorporation of bio-monitoring and health condition data to move beyond exposure to actual health impacts; attention to especially vulnerable populations such as children, seniors, prisoners, and undocumented residents; longitudinal studies to track the relationships between environmental hazards, social vulnerability factors, and health conditions overtime; and methods to track individual mobility and behaviors to better account for the multiple exposure pathways.

## 6. Conclusions and Discussion

Despite the limitations of the CEVA method, it offers clear advantages by analyzing multiple factors involved in environment hazards and social vulnerability. Besides national air toxic assessment, CEVA includes other indicators of localized environmental hazards such as pesticide applications and point source pollutions sites. It goes beyond income and race when considering the social vulnerability of the residents by incorporating formal education, English language fluency, age, and in-patient residence into the model. It also brings in health status as a reference to illustrate how the existing health problems may exacerbate the vulnerability to environmental hazards.

Given the focus of environmental justice on the disproportionate burdens of environmental hazards on the most vulnerable populations and places [16] it can be argued that the CEVA map (Figure 2) reflects the spirit as well as the letter of the relevant laws. We recommend that these areas receive special consideration in permitting, monitoring, and enforcement actions, as well as investments in public participation, capacity building, and community economic development. For example, the Air District may use the CEVA to focus its resources, such as incentive funds, air quality monitoring, permitting reviews, enforcement, and public education and outreach in these most vulnerable areas [45]. The Air District might also use the CEVA to advocate for additional funds from state and federal sources for such enhanced protections of these areas and to track its own progress over time. Beyond the Air District, collaborative efforts that bring together local, regional, state and federal agencies across multiple media (air, water, land) and issue areas (transportation, housing and land use) along with community leaders, will be needed to develop holistic strategies to address cumulative environmental vulnerabilities [46]. 
